# Determination of double- and single-stranded DNA breaks in bovine sperm is predictive of their fertilizing capacity

**DOI:** 10.1186/s40104-022-00754-8

**Published:** 2022-09-17

**Authors:** Jordi Ribas-Maynou, Ariadna Delgado-Bermúdez, Yentel Mateo-Otero, Estel Viñolas, Carlos O. Hidalgo, W. Steven Ward, Marc Yeste

**Affiliations:** 1 Biotechnology of Animal and Human Reproduction (TechnoSperm), Faculty of Sciences, Institute of Food and Agricultural Technology, University of Girona, C/ Maria Aurèlia Campany, 69, ES-17003 Girona, Spain; 2 Unit of Cell Biology, Department of Biology, Faculty of Sciences, University of Girona, ES-17003 Girona, Spain; 3 Institute for Biogenesis Research, Department of Anatomy, Biochemistry & Physiology, John A. Burns School of Medicine, University of Hawaii at Manoa, Honolulu, HI 96822 USA; 4 Department of Animal Selection and Reproduction, The Regional Agri-Food Research and Development Service of Asturias (SERIDA), ES-33394 Gijón, Spain; 5 Catalan Institution for Research and Advanced Studies (ICREA), ES-08010 Barcelona, Spain

**Keywords:** Cattle, Chromatin, Comet test, DNA damage, Fertility, Sperm, Sperm quality

## Abstract

**Background:**

The analysis of chromatin integrity has become an important determinant of sperm quality. In frozen-thawed bovine sperm, neither the sequence of post-thaw injury events nor the dynamics of different types of sperm DNA breaks are well understood. The aim of the present work was to describe such sperm degradation aftermath focusing on DNA damage dynamics, and to assess if this parameter can predict pregnancy rates in cattle.

**Results:**

A total of 75 cryopreserved ejaculates from 25 Holstein bulls were evaluated at two post-thawing periods (0-2 h and 2-4 h), analyzing global and double-stranded DNA damage through alkaline and neutral Comet assays, chromatin deprotamination and decondensation, sperm motility, viability, acrosomal status, and intracellular levels of total ROS, superoxides and calcium. Insemination of 59,605 females was conducted using sperm from the same bulls, thus obtaining the non-return to estrus rates after 90 d (NRR). Results showed an increased rate of double-stranded breaks in the first period (0-2 h: 1.29 ± 1.01%/h vs. 2-4 h: 0.13 ± 1.37%/h; *P* <  0.01), whereas the rate of sperm with moderate + high single-stranded breaks was higher in the second period (0-2 h: 3.52 ± 7.77 %/h vs. 2-4h: 21.06 ± 11.69 %/h; *P* < 0.0001). Regarding sperm physiology, viability decrease rate was different between the two periods (0-2 h: − 4.49 ± 1.79%/h vs. 2-4 h: − 2.50 ± 3.39%/h; *P* = 0.032), but the progressive motility decrease rate was constant throughout post-thawing incubation (0-2 h: − 4.70 ± 3.42%/h vs. 2-4 h: − 1.89 ± 2.97%/h; *P* > 0.05). Finally, whereas no correlations between bull fertility and any dynamic parameter were found, there were correlations between the NRR and the basal percentage of highly-damaged sperm assessed with the alkaline Comet (*Rs* = − 0.563, *P* = 0.003), between NRR and basal progressive motility (*Rs* = 0.511, *P* = 0.009), and between NRR and sperm with high ROS at 4 h post-thaw (*Rs* = 0.564, *P* = 0.003).

**Conclusion:**

The statistically significant correlations found between intracellular ROS, sperm viability, sperm motility, DNA damage and chromatin deprotamination suggested a sequence of events all driven by oxidative stress, where viability and motility would be affected first and sperm chromatin would be altered at a later stage, thus suggesting that bovine sperm should be used for fertilization within 2 h post-thaw. Fertility correlations supported that the assessment of global DNA damage through the Comet assay may help predict bull fertility.

**Supplementary Information:**

The online version contains supplementary material available at 10.1186/s40104-022-00754-8.

## Introduction

Oocyte fertilization by a sperm cell that contains an intact genetic material is crucial to ensure proper embryo development and successful implantation [1–3]. Sperm DNA damage has been previously associated to alterations in sperm quality parameters, such as motility or morphology, and with a reduced fertility potential [4–6]. It is known, however, that although sperm carrying chromatin alterations are sometimes able to fertilize an oocyte, the resulting embryos may bear increased rates of chromosomal alterations and show impaired development [7–9]. While different agents could potentially induce sperm DNA damage, the most important source of this affectation is oxidative stress (OS), which results from the imbalance of the antioxidant/oxidant ratio. Related to this, it is worth mentioning that, because sperm lack transcription and translation activities, the seminal plasma is the main source of antioxidants to scavenge reactive oxygen species (ROS) [10–12]. In fact, the seminal plasma is one of the most antioxidant-rich fluids which, compared to blood, has a 10-fold greater antioxidant potential [13]. The seminal plasma, however, is removed during the process of sperm cryopreservation and, therefore, frozen-thawed samples retain less antioxidant capacity and show little resilience to ROS-related injuries [14].

Reactive oxygen species consist of highly reactive free radicals derived from oxygen that are fundamental for physiological processes such as capacitation, acrosome reaction, sperm hyperactivation or sperm-oocyte interaction [15, 16]. At the same time, however, they are the most damaging factors for sperm DNA, plasma membrane, proteins and other structures [17–19]. These ROS may be synthesized endogenously as a by-product of mitochondria, which generate hydroxyl radicals and superoxides at complexes I and III of the respiratory chain [20, 21], or are exogenous to sperm, like in the case of seminal leucocytes, which may produce up to 1000 times more ROS than a sperm cell [22]. Consequences of a misbalance between antioxidants and ROS include lipid peroxidation, protein modifications and DNA damage. Lipid peroxidation leads to disturbances in membrane fluidity and permeability, causing a reduction of motility and a decrease in the sperm ability to interact and fuse with the oocyte [23]. Even though the injury may affect membranes of any cell type, those of sperm are known to be especially susceptible to oxidative damage due to their great content of polyunsaturated fatty acids [24]. In addition, thiol oxidation by ROS affects the activity of some enzymes, and may also lead to alterations on sperm chromatin, as protamines are rich in thiol groups (cysteine residues) [18, 25]. Furthermore, sperm DNA damage may result from the modification of nitrogenous bases, generating base derivatives such as 8-hydroxy-2′-deoxyguanosine (8OHdG), which, in turn, are transformed into single-stranded DNA breaks (SSB) thanks to the ability of sperm to initiate the very first stage of the base excision repair pathway [23, 26].

Although data available report that ROS affect sperm motility, plasma membrane and DNA integrity, the resilience of bovine sperm to ROS in the next hours after thawing has not been investigated in much detail. Particularly, a previous study analyzing the dynamics of sperm DNA fragmentation through the sperm chromatin dispersion (halo) test after 4 h of incubation in different species, including cattle, showed that the presence of protamine 2 is associated to a higher degree of DNA damage. In species having only protamine 1, a greater number of cysteine residues is related to a higher protection versus DNA damage [27]. Other authors found a negative association between bull fertility and sperm DNA fragmentation evaluated through the halo test, showing that bulls in the <10th percentile of fertility could be identified using this parameter [28]. In spite of this, whether different types of DNA damage (single- and double-stranded), protamination or chromatin condensation in bovine sperm are related to fertilizing capacity has not been investigated. In both artificial insemination and in vitro fertilization using frozen-thawed sperm, oocyte penetration by sperm does not occur immediately after thawing [29, 30]. Because of that, the aim of the present study was to assess how sperm withstand the stress produced by freezing and thawing when incubated up to 4 h post-thaw. Specifically, the present work aimed to evaluate how sperm chromatin integrity, including the incidence of single- and double-stranded breaks, is affected in these processes, and examine whether the chromatin resilience to this post-thawing incubation period is related to sperm function and fertility.

## Materials and methods

All reagents were purchased from Sigma-Aldrich (Saint-Louis, MO, USA), unless otherwise stated.

### Semen samples and study design

The present work included 75 ejaculates from 25 sexually mature (1.5 to 2 years old) Holstein bulls (three ejaculates per animal), collected with a time separation of at least 5 weeks between ejaculates. In order to conduct the experiments described herein, a pool of the three ejaculates was prepared (biological replicate) just after thawing and prior to any determination or analysis of frozen-thawed sperm quality. Briefly, sperm from the three independent ejaculates were mixed, with an equal amount of sperm from each ejaculate. Thereafter, each pool was incubated in a water bath at 38 °C, and the parameters included in the study were measured upon thawing (the 0 h time point was assigned to the sample after thawing but a 10-min period at 38 °C to recover was provided), and after 2 and 4 h. These three time points (i.e., 0, 2 and 4 h) allowed the division of this post-thawing incubation into two periods (first period: from T = 0 h to T = 2 h; and second period: from T = 2 h to T = 4 h). Dynamic parameters were calculated as the rates per hour for the increase or decrease of each variable, and were measured for the two periods.

### Animal housing and semen collection

Animals were housed at the Cenero Artificial Insemination Centre (Gijón, Spain), and were fed a standard diet adjusted to bull studs. The center complied with all European Union regulations for animal husbandry, and because samples used in the present study were collected for commercial purposes and authors did not conduct any intervention, no approval from an Ethics Committee was required.

Sperm samples were collected by an experienced technician. Bulls were allowed to false mounting to become stimulated and semen was collected through an artificial vagina, with an internal temperature of 45 °C. Following this, semen samples were evaluated using a commercial computer-assisted system (Integrated Sperm Analysis System V1.0; Proiser S.L., Valencia, Spain), following the procedure described in Sperm motility section. Cryopreservation was only performed in non-contaminated ejaculates with ≥10^9^ sperm/mL and ≥ 85% of total motile sperm, following the protocol described below (Sperm equilibration and cryopreservation section).

### Sperm equilibration and cryopreservation

Initially, samples were equilibrated in a commercial extender at 22 °C (Bioxcell; IMV Technologies, L’Aigle, France), adjusting the concentration to 92 × 10^6^ sperm/mL. Cryopreservation was conducted following a standard procedure described previously [31]. The mixture was then cooled to 4 °C at a rate of − 0.2 °C/min, and equilibrated at this temperature for 3 h. At this point, sperm were packed into 0.25-mL straws, and the following cooling rates were applied using a controlled-rate freezer (Digit-Cool; IMV Technologies): 5 °C/min from 4 °C to − 10 °C; 40 °C/min from − 10 °C to − 100 °C; and 20 °C/min from − 100 °C to − 140 °C. Finally, straws were stored in liquid nitrogen until use. Thawing was performed through immersion of straws at 38 °C for 20 s in a water bath.

### Artificial insemination

Artificial insemination (AI) was conducted using frozen straws produced from the ejaculates utilized in this study and from other ejaculates collected from the same 25 bulls. Average fertility rates were obtained through the proportion of females that did not return to estrus after 90 d of insemination (NRR). The total number of animals inseminated was 59,605, and the average number of inseminated females per bull was 2384 (minimum: 207; maximum: 15,231).

### Evaluation of global and double-stranded sperm DNA fragmentation: alkaline and neutral Comet assays

The Comet assay was used to determine sperm DNA damage in each of the 25 biological replicates. The incidence of double-stranded breaks (DSB) was determined through the neutral Comet assay, whereas the total amount of DNA breaks including single- and double-stranded DNA damage (global DNA damage) was determined through the alkaline Comet assay. The protocol applied for both variants was that described elsewhere [32] and adapted to bovine sperm, with a previous step to ensure complete chromatin decondensation (see Slide pretreatment, sample preparation, agarose embedding and lysis section).

#### Slide pretreatment, sample preparation, agarose embedding and lysis

Previous to the initiation of the protocol, pretreated slides were prepared by adding a thin layer of low melting point agarose 1% dissolved in distilled water, allowing the slides to dry overnight.

First, an aliquot of sperm cells at 5 × 10^5^ sperm/mL was mixed 1:2 (v:v) with an aliquot of melted low melting point agarose dissolved in TBE buffer (90 mmol/L Tris-base, 90 mmol/L Boric acid, 2 mmol/L EDTA, pH 8), warmed at 37 °C, reaching a final agarose concentration of 0.66%. Two drops of 6.5 μL were placed onto two different agarose-pretreated slides, one for alkaline and the other for neutral Comet assays, respectively. Rapidly, a round coverslip was used to cover samples, which were allowed to jellify on a metal cold plate at 4 °C for 5 min. Then, coverslips were removed and samples were incubated with three lysis solutions, following the recommendations of a previous work that showed that a long third lysis solution is required for complete chromatin decondensation in bovine sperm [33]. Hence, slides were incubated in the first lysis solution (0.8 mol/L Tris-HCl, 0.8 mol/L DTT, 1% SDS, pH 7.5) for 30 min, in the second lysis solution (0.4 mol/L Tris-HCl, 0.4 mol/L DTT, 50 mmol/L EDTA, 2 mol/L NaCl, 1% Tween20, pH 7.5) for 30 min, and in the third lysis solution (0.4 mol/L Tris-HCl, 0.4 mol/L DTT, 50 mmol/L EDTA, 2 mol/L NaCl, 1% Tween20, 100 μg/mL Proteinase K, pH 7.5) for 180 min.

#### Electrophoresis

Alkaline and neutral Comet assays were conducted at different electrophoresis conditions. For neutral Comet, electrophoresis was conducted in TBE buffer at 1 V/cm for 30 min, and slides were then washed in 0.9% (w/v) NaCl for 2 min. For alkaline Comet, a previous DNA denaturation step was conducted in an alkaline solution containing 0.03 mol/L NaOH and 1 mol/L NaCl at 4 °C for 5 min, and electrophoresis was performed in an alkaline buffer (0.03 mol/L NaOH; pH 13) at 1 V/cm for 4 min.

#### Neutralization, dehydration and staining

At this stage, the pH of samples was neutralized by incubating slides in a 0.4 mol/L Tris-HCl solution at pH = 7.5 for 5 min. Samples were then fixed in a 70%-90%-100% ethanol series for 2 min each, and finally dried in horizontal position. Staining was conducted through immersion of slides in 1× Safeview DNA staining solution (NBS Biologicals, Huntingdon, UK) for 15 min and allowed to dry horizontally.

#### Imaging and analysis

Comet preparations were visualized under an epifluorescence microscope (Zeiss Imager Z1, Carl Zeiss AG, Oberkochen, Germany) equipped with the filter set 40 (Zeiss Imager Z1, Carl Zeiss AG, Oberkochen, Germany), which allows excitation wavelengths of 360/51, 485/17 and 560/18, and emission wavelengths of 460 nm, 520 nm and 600 nm. At least 150 sperm were captured at 1000× magnification and 1388 × 1040 resolution using the Axiovision 4.6 software (Carl Zeiss AG, Oberkochen, Germany), adjusting the exposure time in each field to avoid overexposure.

Fluorescence intensity of Comet heads and tails was analyzed through the CometScore v2.0 software [34], using the automatic function of the software, which automatically identifies Comet shapes. After automatic analysis, a manual review of each image was conducted to eliminate captures not corresponding to comets, to eliminate overlapping comets, and to correct misidentifications of Comet heads and tails. A low limit of 50 analyzable comets was established as a minimum to measure DNA damage intensity, and more pictures were captured and analyzed when this figure was not reached.

#### Determination of DNA damage intensity and percentages of sperm with fragmented DNA

Data files including Comet head intensity (arbitrary units (AU)), tail intensity (AU), tail length (pixels) and percentage of DNA in tail (Tail DNA) were extracted from CometScore as .csv; Microsoft Excel (Microsoft, Redmond, WA, USA), and Statistics Package for Social Sciences ver. 25.0 (IBM Corporation; Armonk, NY, USA) were used to calculate the intensity of DNA damage and the percentage of sperm with fragmented DNA.

To quantify the amount of DNA breaks (DNA damage intensity), the Olive Tail Moment (OTM), calculated as [(Tail mean intensity – Head mean intensity) × Tail DNA/100] was used.

In order to obtain the percentage of sperm with double-stranded DNA fragmentation (%dsSDF) and the percentage of sperm with global DNA damage (%SDF), a principal component analysis (PCA) was run using Tail Length, Tail DNA and Olive Tail Moment from alkaline and neutral Comets, independently. All three parameters were sorted into a single component, which was used to calculate regression scores, assigned to each analyzed cell. These regression scores were utilized as input variables in a cluster analysis to classify each sample using the between-groups linkage method, based on the Euclidean distance. Three different subpopulations for the alkaline Comet, corresponding to sperm with high, moderate and low incidence of DNA breaks; and two different subpopulations for the neutral Comet, corresponding to high and low incidence of double stranded breaks (DSB), were identified.

### Evaluation of sperm quality

#### Sperm motility

Sperm motility and kinematic parameters were assessed at 0, 2 and 4 h post-thaw using a commercial computer-assisted system (Integrated Sperm Analysis System V1.0; Proiser S.L., Valencia, Spain). Five μL of each sample were loaded into a previously warmed (38 °C) 20-μm Leja chamber slide (Leja Products BV; Nieuw-Vennep, The Netherlands). Two technical replicates were examined, analyzing 1000 spermatozoa per replicate (at least three different fields) at 100× magnification (Olympus BX41; Olympus, Tokyo, Japan). Percentages of total and progressive motility were recorded for each sample. Sperm were considered motile when their average path velocity (VAP) was ≥10 μm/s, and progressively motile when their straightness index (STR) was ≥70%. The following kinematic parameters were also recorded: velocity (fast, medium or slow); curvilinear velocity (VCL, μm/s); straight-line velocity (VSL, μm/s); average path velocity (VAP, μm/s); linearity (LIN = VSL/VCL × 100, %); straightness (STR = VSL/VAP × 100, %); oscillation (WOB = VAP/VCL × 100, %); lateral head displacement (ALH, μm); and frequency of head displacement (BCF, Hz).

#### Sperm morphology

Sperm morphology was assessed after incubation in 2% formaldehyde at room temperature for 5 min. Briefly, 5 μL of this mixture was poured onto a glass slide and covered with a 20 × 20 coverslip. Two replicates including at least 100 sperm per replicate were examined, and the percentage of sperm with abnormal morphology (including sperm with cytoplasmic droplets, head abnormalities, tail abnormalities, or isolated heads) was calculated.

### Flow cytometry

Flow cytometry parameters described below were analyzed using a CytoFLEX flow cytometer (Beckman Coulter, Fullerton, CA, USA), equipped with red, blue and violet lasers (637, 488 and 405 nm). All incubations with fluorescent probes were conducted in samples adjusted to 1 × 10^6^ sperm/mL in pre-warmed PBS, and two replicates were examined evaluating 10,000 sperm per replicate (flow rate was between 10 μL/s and 60 μL/s). Analysis of flow cytometry dot-plots was conducted through the CytExpert Software (Beckman Coulter; Fullerton, CA, USA).

Fluorochromes were purchased from ThermoFisher (Waltham, MA, USA). Hydroethidine (HE) was excited with the 488-nm laser and detected through the PE channel (585/42). SYBR-14, YO-PRO-1, Fluo-3 and H_2_DCFDA were excited with the 488-nm laser and detected with the FITC channel (525/40). Propidium iodide (PI) was excited with the 488-nm laser and detected through the PC5.5 channel (690/50). Chromomycin (CMA3) was excited with the 405-nm laser and its fluorescence was collected with the Violet610 channel (610/20).

#### Sperm viability

Sperm viability was determined by staining samples with SYBR-14 (32 nmol/L) and PI (7.5 μmol/L) for 15 min at 38 °C. Sperm stained in green and gated in SYBR-14^+^/PI^−^ were considered as viable. The final percentage of viable sperm was recorded after subtracting the debris (SYBR-14^−^/PI^−^).

#### Intracellular superoxide radicals (Hydroethidine, HE)

Hydroethidine is a compound that oxidizes into Ethidium (E) in the presence of O_2_^−^. In order to determine intracellular superoxide levels, samples were incubated in HE (5 μmol/L) and YO-PRO-1 (31.25 nmol/L) for 20 min at 38 °C. Fluorescence emitted by E^+^ that spilled into the FITC channel was compensated (2.24%), as too was that from YO-PRO-1 that spilled into the PE channel (7.5%). After analysis, four subpopulations could be identified: viable sperm with low levels of superoxides (E^−^/YO-PRO-1^−^); viable sperm with high levels of superoxides (E^+^/YO-PRO-1^−^); non-viable sperm with low levels of superoxides (E^−^/YO-PRO-1^+^); and non-viable sperm with high levels of superoxides (E^+^/YO-PRO-1^+^).

#### Intracellular reactive oxygen species (H_2_DCFDA)

The 2′,7′-dichlorodihydrofluorescein diacetate (H_2_DCFDA) is a cell-permeant compound that, in the presence of reactive oxygen species, is converted into dichlorofluorescein (DCF), emitting green fluorescence. Intracellular ROS was evaluated after staining with H_2_DCFDA (100 μmol/L) and PI (5.6 μmol/L) for 20 min at 38 °C. After analysis, four subpopulations could be identified: viable spermatozoa with low levels of ROS (DCF^−^/PI^−^); viable sperm with high levels of ROS (DCF^+^/PI^−^); non-viable sperm with low levels of ROS (DCF^−^/PI^+^); and non-viable sperm with high levels of ROS (DCF^+^/PI^+^).

#### Intracellular calcium (Fluo3)

Fluo3 is a cell-permeant indicator that exhibits an increase of fluorescence upon Ca^2+^ binding. For the measurement of intracellular calcium, samples were incubated with Fluo3 (1.17 μmol/L) and PI (5.6 μmol/L) for 10 min at 38 °C. After analysis, four subpopulations were identified: viable sperm with low levels of Ca^2+^ (Fluo3^−^/PI^−^); viable sperm with high levels of Ca^2+^ (Fluo3^+^/PI^−^); non-viable sperm with low levels of Ca^2+^ (Fluo3^−^/PI^+^); and non-viable sperm with high levels of Ca^2+^ (Fluo3^+^/PI^+^).

### Evaluation of chromatin status

#### Sperm protamination (Chromomycin A3, CMA3)

Chromomycin A3 is a glycoside antibiotic that, in the presence of Mg^2+^, binds to DNA and emits fluorescence. In sperm cells, this DNA-ability is only possible in regions with poor protamination, so that CMA3 is used as an indicator of chromatin (de)protamination. Briefly, samples at a concentration of 20 × 10^6^ sperm/mL were diluted 1:1 (v:v) in 2× McIlvine solution (60 mmol/L citric acid, 280 mmol/L Na_2_HPO_4_ and 20 mmol/L MgCl_2_) containing 12.5 μg/mL CMA3, and incubated for 30 min at room temperature. Subsequently, samples were diluted 1:10 (v:v) in PBS, and examined with a flow cytometer according to the parameters described above (Flow cytometry section). The fluorescence intensity for Violet610 channel was recorded as a quantitative measure of deprotamination degree. A negative control without CMA3 was conducted in parallel to establish the threshold value for positive CMA3 cells, leading to the final identification of two sperm populations: CMA3^+^ and CMA3^−^. The percentage of sperm with a high degree of deprotamination (CMA3^+^) was recorded.

#### Sperm chromatin decondensation susceptibility (neutral halo assay)

The sperm chromatin dispersion test (halo assay) assesses the susceptibility of sperm chromatin to decondense. The halo assay was conducted following complete chromatin decondensation as described previously [33]. In brief, sperm were diluted to 2 × 10^6^ and mixed 1:2 (v:v) with low melting point agarose at 37 °C. Then, 6.5 μL of the mixture was poured onto an agarose-treated slide, covered with a coverslip, and allowed to jellify for 5 min on the top of a metal plate at 4 °C. After removing coverslips, samples were incubated with three lysis solutions: first lysis (0.8 mol/L Tris-HCl, 0.8 mol/L DTT, 1% SDS, pH 7.5) for 30 min, second lysis (0.4 mol/L Tris-HCl, 0.4 mol/L DTT, 50 mmol/L EDTA, 2 mol/L NaCl, 1% Tween20, pH 7.5) for 30 min, and third lysis (0.4 mol/L Tris-HCl, 0.4 mol/L DTT, 50 mmol/L EDTA, 2 mol/L NaCl, 1% Tween20, 100 μg/mL Proteinase K, pH 7.5) for 180 min. Finally, slides were washed in distilled water, dehydrated in an ethanol series (70%, 90% and 100%) for 2 min each, and dried horizontally. The analysis of decondensed sperm was conducted following the criteria shown in Additional file 1 (Fig. S1), which were based on Fernández et al. [35].

### Statistical analyses

Statistical analysis was conducted with IBM SPSS 27.0 for Windows (IBM Corp.; Armonk, NY, USA) and graphs were prepared using GraphPad Prism v8 (GraphPad Software, La Jolla, CA, USA). First, normal distribution and homogeneity of variances were tested with Shapiro-Wilk and Levene tests, respectively. If parameters did not fit with parametric assumptions and this was not remedied after linear transformation (arcsin √x, √x), non-parametric tests were used as an alternative.

Differences between time points (0, 2, and 4 h) were evaluated through a repeated measures one-way ANOVA or Friedman test followed by Dunn’s post-hoc test for pair-wise comparisons. For multiple comparisons, a two-way ANOVA followed by Tukey post-hoc test was conducted. Correlations were assessed through the Spearman test.

Each pool of samples from a given bull was considered as a statistical case. For all tests, the level of statistical significance was set at *P* ≤ 0.05.

## Results

### Sperm DNA damage

#### Double-stranded DNA breaks

The incidence of double-stranded DNA breaks (Olive Tail Moment) and the percentage of sperm with double-stranded DNA fragmentation (%dsSDF) obtained at the different time points are shown in Table 1. While a small increase for both the incidence of DNA breaks and the percentage of sperm with double-stranded breaks (%dsSDF) was observed during post-thawing incubation, only the latter was statistically significant (*P <* 0.001).

**Table 1:** Effects of post-thawing incubation time on single- and double-stranded sperm DNA damage

Items	T = 0 h	T = 2 h	T = 4 h	*P-*value
	Mean ± SD	Mean ± SD	Mean ± SD	
Neutral Comet				
dsDNA OTM				
Neutral Comet OTM, AU	1.31 ± 0.20	1.44 ± 0.27	1.48 ± 0.29	n.s.
%dsSDF				
Low DNA damage, %	99.96 ± 0.20	97.38 ± 1.95^*^	97.11 ± 3.25^*^	< 0.0001
High DNA damage, %	0.04 ± 0.20	2.62 ± 1.95^*^	2.89 ± 3.25^*^	< 0.0001
Alkaline Comet				
ssDNA+dsDNA OTM				
Alkaline Comet OTM, AU	15.57 ± 5.20	16.80 ± 5.59	28.38 ± 7.82^*#^	< 0.0001
%ssDNA+dsDNA				
Low DNA damage, %	74.12 ± 24.21	67.09 ± 27.04	24.97 ± 22.12^*#^	< 0.0001
Moderate DNA damage, %	25.05 ± 22.93	26.42 ± 21.20	55.41 ± 18.55^*#^	< 0.001
High DNA damage, %	0.83 ± 2.07	6.49 ± 10.46^*^	19.62 ± 20.47^*#^	< 0.0001
Moderate + high DNA damage, %	25.88 ± 24.21	32.91 ± 27.04	75.03 ± 22.12^*#^	< 0.0001

*OTM* Olive tail moment, indicating DNA breaks intensity, *%dsSDF* Percentage of sperm with double-stranded DNA fragmentation, *%ssSDF* Percentage of sperm with single-stranded DNA fragmentation, *n.s.* non-statistically significant differences

^*^Differences compared to 0 h (*P* < 0.01)

^#^Differences compared to 2 h (*P* < 0.01)

**Fig. 1:**
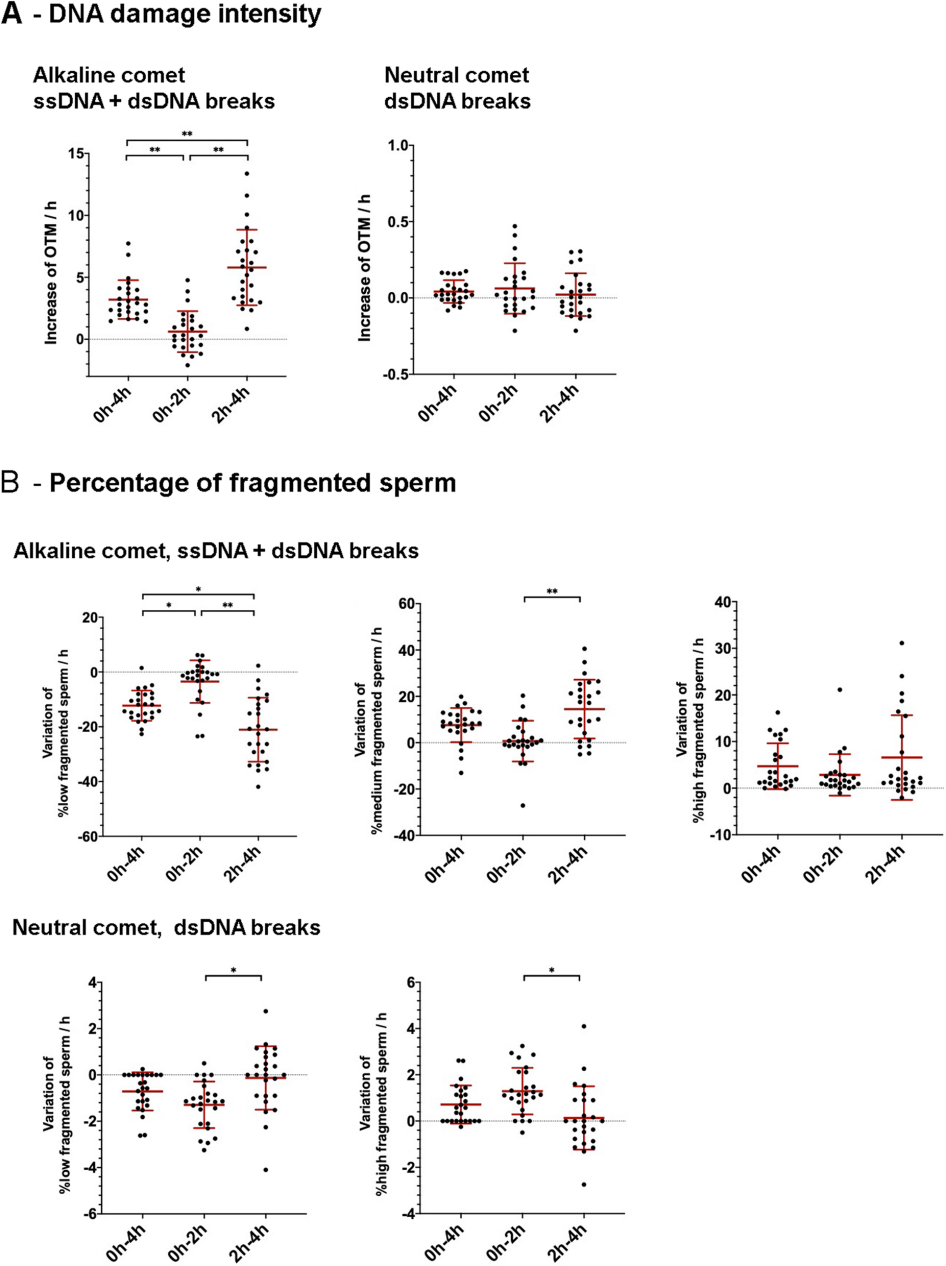
Rates of increase or decrease between the two periods for **A** the extent of DNA damage, and **B** the percentage of sperm with fragmented DNA, evaluated with alkaline and neutral Comet assays

The increase in the incidence of double-stranded DNA damage was similar between the first (from 0 to 2 h) and the second period (from 2 to 4 h; Fig. 1A). The rate of increase for the percentage of sperm with double-stranded DNA fragmentation (%dsSDF), however, was significantly (*P* < 0.01) higher in the first (1.29 ± 1.01%/h) than in the second period (0.13 ± 1.37%/h; Fig. 1B).

#### Global DNA damage

Results of the assessment of global DNA damage are depicted in Table 1. A significant increase over post-thawing incubation, both for the amount of DNA breaks and for the percentage of sperm with moderate or high DNA fragmentation, was observed *(P* < 0.0001).

The rate of increase for the incidence of sperm DNA damage differed between the first and second periods (0.613 ± 1.65 AU/h vs. 5.79 ± 3.05 AU/h; *P* <  0.001; Fig. 1A). Similarly, the rate of decrease for the percentage of sperm with low DNA fragmentation differed between these two periods (− 3.52 ± 7.77%/h vs. −21.06 ± 11.69%/h; *P* < 0.0001; Fig. 1B), and the increase rate for the percentage of sperm with moderate DNA fragmentation showed statistically significant differences (0.68 ± 8.81%/h vs. 14.50 ± 12.69%/h; *P* < 0.001; Fig. 1B). The rate of increase for the percentage of sperm with high DNA fragmentation did not present statistically significant differences (2.83 ± 4.46%/h vs. 6.56 ± 9.09%/h; *P* = 0.141; Fig. 1B).

### Sperm chromatin status

#### Chromatin decondensation

**Fig. 2:**
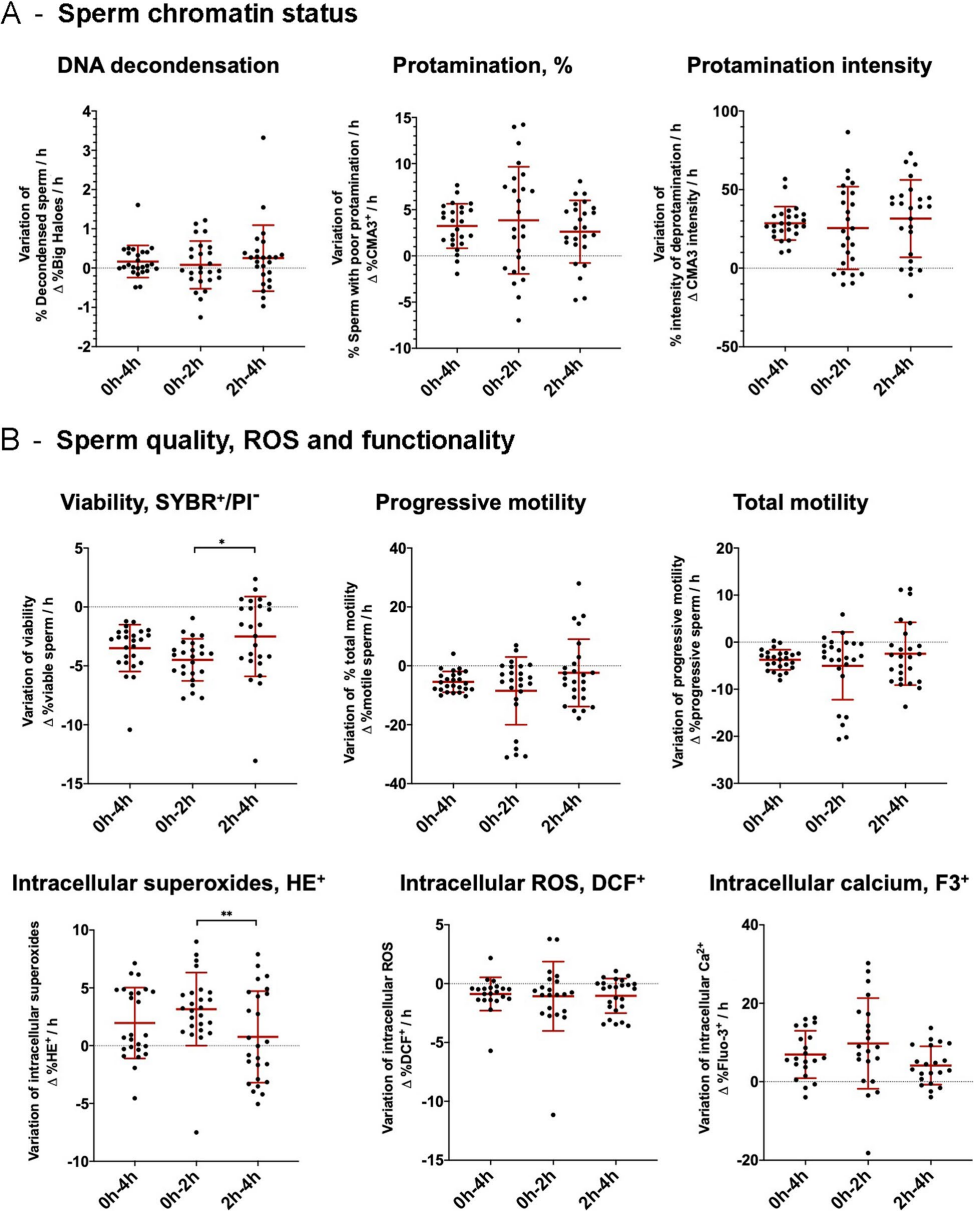
Rates of increase or decrease between the two periods for **A** sperm chromatin status, and **B** sperm quality, ROS and functionality parameters

Although the percentage of sperm with decondensed chromatin increased slightly over post-thawing incubation, no statistical differences between time points were observed (*P* > 0.05) (Table 2). The rates of increase for sperm chromatin decondensation, which were 0.08 ± 0.60%/h and 0.25 ± 0.83%/h, for the first (0 to 2 h) and second (2 to 4 h) periods, respectively, were not statistically different from each other (*P* > 0.05; Fig. 2).

**Table 2:** Effects of post-thawing incubation time on sperm chromatin parameters

Items	T = 0 h	T = 2 h	T = 4 h	*P*-value
	Mean ± SD	Mean ± SD	Mean ± SD	
DNA decondensation (halo test)				
Sperm with increased DNA decondensation, %	2.87 ± 1.56	3.03 ± 1.06	3.53 ± 1.78	n.s.
Sperm protamination (CMA3)				
Intensity of chromatin deprotamination, AU	802.04 ± 41.03	853.12 ± 36.20^*^	916.18 ± 35.83^*#^	< 0.0001
Sperm with poor protamination, %CMA3^+^	16.76 ± 10.07	24.47 ± 7.68^*^	29.71 ± 6.61^*#^	< 0.0001

*n.s.* non-statistically significant differences

^*^Differences compared to 0 h (*P* < 0.05)

^#^Differences compared to 2 h (*P* < 0.05)

#### Sperm protamination

Two measures of sperm protamination were made: the degree of deprotamination and the percentage of affected sperm cells. Both parameters showed a statistically significant increase after both 2 and 4 h of incubation (*P* < 0.001) (Table 2). The rates of increase for the deprotamination degree were 25.54 ± 26.32 AU/h and 31.53 ± 22.66 AU/h, and those for the proportions of affected sperm cells were 3.85 ± 5.81%/h and 2.62 ± 3.39%/h, for the two periods, respectively. No significant differences between these rates were observed (*P* > 0.05) (Fig. 2).

### Sperm quality, oxidative stress and sperm function

#### Viability

Sperm viability suffered a statistically significant reduction throughout post-thawing incubation time (*P* < 0.001) (Fig. 2B). The rates of that reduction were not equal between periods (*P* = 0.032), as sperm survival decreased more quickly in the first (0 to 2 h; − 4.49 ± 1.79%/h) than in the second period (2 to 4 h; − 2.50 ± 3.39%/h) (Fig. 2).

**Table 3:** Effects of post-thawing incubation time on sperm morphology, viability, motility and kinematic parameters

Items	T = 0 h	T = 2 h	T = 4 h	*P*-value
	Mean ± SD	Mean ± SD	Mean ± SD	
Morphology				
Normal morphology, %	88.97 ± 3.22			
Abnormal morphology, %	11.05 ± 3.22			
Head abnormalities	0.85 ± 0.80			
Cytoplasmic droplets	0.64 ± 0.76			
Tail abnormalities	6.83 ± 2.93			
Isolated heads	2.72 ± 1.61			
Viability				
Viable sperm, %	50.95 ± 9.55	41.97 ± 9.03^*^	36.96 ± 10.76^*^	< 0.0001
Motility				
Progressive Motility, %	27.96 ± 9.35	22.35 ± 9.36^*^	12.96 ± 9.02^*#^	< 0.0001
Non-progressive Motility, %	19.92 ± 7.63	16.23 ± 7.62^*^	13.13 ± 9.30^*^	< 0.05
Total Motility, %	47.89 ± 14.20	38.58 ± 15.55^*^	26.09 ± 16.94^*#^	< 0.001
Kinematic parameters				
Fast velocity, %	31.00 ± 11.48	24.22 ± 11.32^*^	12.62 ± 10.10^*#^	< 0.001
Medium velocity, %	8.89 ± 2.74	8.48 ± 2.98	8.30 ± 4.16	n.s.
Slow velocity, %	8.76 ± 7.97	5.88 ± 4.13^*^	5.16 ± 5.57^*^	< 0.05
VCL, μm/s	81.97 ± 16.29	73.22 ± 13.23^*^	51.96 ± 13.99^*#^	< 0.01
VSL, μm/s	43.68 ± 13.07	37.63 ± 10.61	22.39 ± 10.39^*#^	< 0.001
VAP, μm/s	50.10 ± 13.09	43.68 ± 10.63^*^	28.42 ± 10.17^*#^	< 0.01
LIN, %	52.37 ± 6.82	50.38 ± 7.20	41.08 ± 9.37^*#^	< 0.01
STR, %	86.26 ± 4.79	85.07 ± 5.73	75.76 ± 10.60^*#^	< 0.01
WOB, %	60.49 ± 5.35	58.92 ± 5.29	53.61 ± 5.59^*#^	< 0.01
ALH, μm	3.11 ± 0.49	2.77 ± 0.42^*^	2.17 ± 0.52^*#^	< 0.01
BCF, Hz	12.28 ± 1.36	11.99 ± 1.76	8.80 ± 3.65^*#^	< 0.01

*n.s.* non-statistically significant differences

^*^Differences compared to 0 h (*P* < 0.05)

^#^Differences compared to 2 h (*P* < 0.05)

#### Motility and kinematic parameters

Progressive motility, non-progressive motility and total motility declined as time advanced (*P* < 0.01). For kinematic parameters, a similar trend was observed after 4 h of incubation, when all parameters suffered a reduction compared to 0 h post-thawing (*P* < 0.01) (Table 3).

For progressive motility, the rates of decreases were − 4.70 ± 3.42%/h and − 1.89 ± 2.97%/h for the first and second periods, respectively (*P* > 0.05) (Fig. 2); for total motility, values were − 7.13 ± 5.95%/h and − 3.52 ± 5.09%/h, for the first and second periods, respectively (*P* > 0.05) (Fig. 2); for sperm with fast velocity, values were − 2.34 ± 3.48%/h and − 6.31 ± 4.39%/h, for the first and second periods (*P* > 0.05).

#### Intracellular ROS

The proportions of sperm with high levels of total ROS (DCF^+^) were reduced over post-thawing incubation; yet, this reduction was only significant after 4 h of incubation (*P* < 0.01) (Table 4). The rate of this decrease was similar between these time periods (0 to 2 h: − 1.08 ± 2.95%/h; 2 to 4 h: − 1.04 ± 1.47%/h; *P* > 0.05) (Fig. 2).

**Table 4:** Effects of post-thawing incubation time on sperm functionality parameters at different evaluation times

Items	T = 0 h	T = 2 h	T = 4 h	*P*-value
	Mean ± SD	Mean ± SD	Mean ± SD	
Intracellular superoxides				
Sperm with high superoxides, %E^+^	47.94 ± 9.09	54.26 ± 8.51^*^	55.78 ± 9.33^*^	< 0.01
Viable sperm with high superoxides, %E^+^/YO-PRO-1^−^	4.04 ± 2.87	3.03 ± 1.60	0.85 ± 0.62^*#^	< 0.01
Viable sperm with low superoxides, %E^−^/YO-PRO-1^−^	47.31 ± 8.78	43.31 ± 8.18^*^	41.60 ± 9.08^*^	< 0.01
Non-viable sperm with high superoxides, %E^+^/YO-PRO-1^+^	43.91 ± 8.75	51.23 ± 8.83^*^	54.93 ± 9.56^*^	< 0.01
Non-viable sperm with low superoxides, %E^−^/YO-PRO-1^+^	4.75 ± 3.78	2.43 ± 0.57^*^	2.62 ± 0.75^*^	< 0.05
Intracellular ROS				
Sperm with high ROS, %DCF^+^	6.13 ± 4.83	4.51 ± 3.24	2.44 ± 2.31^*#^	< 0.01
Viable sperm with high ROS, %DCF^+^/PI^−^	5.61 ± 4.79	3.96 ± 3.24	1.91 ± 2.30^*#^	< 0.01
Viable sperm with low ROS, %DCF^−^/PI^−^	44.84 ± 10.31	36.69 ± 9.58^*^	33.91 ± 12.98^*^	< 0.0001
Non-viable sperm with high ROS, %DCF^+^/PI^+^	0.52 ± 0.09	0.55 ± 0.08	0.53 ± 0.08	n.s.
Non-viable sperm with low ROS, %DCF^−^/PI^+^	49.03 ± 10.82	58.79 ± 8.90^*^	63.65 ± 13.23^*#^	< 0.0001
Intracellular calcium				
Sperm with high intracellular calcium, %F3^+^	59.66 ± 18.09	79.00 ± 10.28^*^	89.26 ± 9.27^*#^	< 0.01
Viable sperm with high intracellular calcium, %F3^+^/PI^−^	13.46 ± 13.84	23.06 ± 11.60	27.88 ± 14.50^*#^	< 0.05
Viable sperm with low intracellular calcium, %F3^−^/PI^−^	37.42 ± 16.94	20.07 ± 9.92^*^	10.19 ± 9.02^*#^	< 0.001
Non-viable sperm with high intracellular calcium, %F3^+^/PI^+^	46.20 ± 9.69	55.94 ± 8.49^*^	61.38 ± 10.70^*#^	< 0.001
Non-viable sperm with low intracellular calcium, %F3^−^/PI^+^	2.92 ± 2.37	0.93 ± 0.83^*^	0.56 ± 0.39^*^	< 0.001

*n.s.* non-statistically significant differences

^*^Differences compared to 0 h (*P* < 0.05)

^#^Differences compared to 2 h (*P* < 0.05)

#### Intracellular superoxides

The percentage of sperm with high levels of intracellular superoxides (HE^+^) increased after 2 h and after 4 h of thawing (*P* < 0.01) (Table 4). This increase was observed to occur at a higher rate during the first (3.16 ± 3.16%/h) compared to the second period (0.76 ± 3.96%/h) (*P* < 0.001) (Fig. 2).

#### Intracellular calcium

Sperm with high intracellular calcium (F3^+^) increased significantly over post-thawing incubation (i.e., 2 and 4 h after thawing; *P* < 0.01) (Table 4), and the rate of increase for this parameter was similar in the two periods (0 to 2 h: 9.75 ± 11.57%/h; 2 to 4 h: 5.13 ± 5.26%/h; *P* > 0.05) (Fig. 2).

### Correlation between the increase/decrease of global and double-stranded sperm DNA damage and other sperm quality parameters

Correlations between basal and dynamic values of DNA damage (global and double-stranded), sperm chromatin condensation, and sperm quality, ROS and functionality parameters are shown in Additional files 2 (Table S1), 3 (Table S2) and 4 (Table S3).

#### Correlations considering the entire post-thawing incubation time (0–4 h)

Focusing on the evolution of the different parameters along the 4 h of incubation, the following statistically significant correlations were observed: i) a positive correlation between the rate of increase for the percentage of sperm with moderate or high degree of DNA fragmentation evaluated though the alkaline Comet and the rate of increase for the percentage of sperm with total ROS (DCF^+^) (*Rs* = 0.604; *P* = 0.004); ii) a positive correlation between the increase of alkaline DNA breaks and the increase of the percentage of sperm with decondensed DNA (*Rs* = 0.430; *P* = 0.032); iii) a negative correlation between the degree of basal DNA damage and the rate of increase for sperm with intracellular calcium (F3^+^) (*Rs* = − 0.460; *P* = 0.036); and iv) a negative correlation between the percentage of sperm with global DNA damage (moderate + high) and the rate of increase for the proportions of sperm with high intracellular calcium (F3^+^) (*Rs* = − 0.512; *P* = 0.018).

No correlation between the rate of increase/decrease for the double-stranded DNA damage and sperm chromatin decondensation, or sperm quality and functionality parameters over the entire post-thawing incubation period was observed (*P >* 0.05).

#### Correlations considering the first period of incubation (0–2 h)

During the first period of incubation, positive correlations between the rate of increase for the percentage of sperm with moderate and high global DNA damage and the variation rates for progressive (*Rs* = 0.484; *P* = 0.036) and total motility (*Rs* = 0.475; *P* = 0.040) were observed. The percentage of sperm with progressive motility at 0 h correlated positively with the proportion of sperm with high intracellular ROS (DCF^+^) (*Rs* = 0.519; *P* = 0.016). In addition, the percentage of sperm with global DNA damage (moderate+high) was negatively correlated with that of progressive motility at 2 h (*Rs* = − 0.459; *P* = 0.042); and positively correlated with the percentage of sperm with high superoxide content (E^+^) at 2 h post-thaw (*Rs* = 0.543; *P* = 0.005). Finally, a positive correlation between the rate of increase of DNA decondensation and that of the degree of double-stranded breaks (*Rs* = 0.477; *P* = 0.016), and a negative correlation between the basal value of double-stranded breaks at 0 h and progressive motility (*Rs* = − 0.473; *P* = 0.041) were found.

#### Correlations considering the second period of incubation (2–4 h)

During the second period of incubation, a positive correlation between the rate of increase for global DNA damage and that for the degree of chromatin deprotamination was observed (*Rs* = 0.607; *P* = 0.001). Moreover, the rate of increase for the percentage of sperm with global DNA damage (moderate + high) was positively correlated with that for the percentage of sperm with decondensed DNA (*Rs* = 0.468; *P* = 0.018) and for the degree of chromatin deprotamination (*Rs* = 0.425; *P* = 0.034). Finally, a negative correlation between the rates of increase for global DNA damage and for the percentage of sperm with high intracellular superoxides (E^+^) was observed (*Rs* = − 0.415; *P* = 0.039).

### Correlation with fertility outcomes after artificial insemination

After assessing sperm quality, all static and dynamic parameters were tested for correlation with non-return rates after artificial insemination. Parameters for which correlations were found to be significant are listed in Table 5. Interestingly, no dynamic parameter correlated to non-return rates (*P* > 0.05). In spite of this, the percentage of sperm with high DNA fragmentation evaluated through the alkaline Comet at 0 h (static measurement) was correlated with 90-d NRR (*Rs* = − 0.563, *P* = 0.003). Moreover, correlations with fertility rates were found for progressive motility at 0 h (*Rs* = 0.511, *P* = 0.009), fast velocity at 0 h *(Rs* = 0.489, *P* = 0.013), progressive motility at 2 h *(Rs* = 0.509, *P* = 0.022), total motility at 2 h *(Rs* = 0.512, *P* = 0.021), fast velocity at 2 h *(Rs* = 0.469, *P* = 0.037), total sperm with high intracellular ROS at 4 h (*Rs* = 0.553, *P* = 0.004) and viable sperm with high intracellular ROS at 4 h (*Rs* = 0.564, *P* = 0.003) (Table 5).

**Table 5:** Statistically significant correlations between the dynamic and static parameters and 90-d NRR

Items	*Rs*	95% Confidence interval	*P*-value
Dynamic parameters			
No statistically significant correlations			
Static parameters			
T0 Alkaline Comet, % High SDF	− 0.563	−0.788 to − 0.204	0.003
T0 Progressive Motility, %	0.511	0.133 to 0.759	0.009
T0 Fast velocity, %	0.489	0.104 to 0.746	0.013
T2 Progressive motility, %	0.509	0.072 to 0.782	0.022
T2 Total motility, %	0.512	0.076 to 0.783	0.021
T2 Fast velocity, %	0.469	0.018 to 0.760	0.037
T4 Sperm with high ROS, %DCF+	0.553	0.189 to 0.782	0.004
T4 Viable sperm with high ROS, DCF+/PI^-^	0.564	0.204 to 0.788	0.003

## Discussion

Understanding how DNA damage occurs in sperm cells before they reach the oocyte is necessary to prevent fertilization failure and develop new strategies aimed to prevent post-thawing sperm deterioration. Herein, we focused on the increase in the extent of global DNA damage and double-stranded DNA breaks after freeze-thawing. Specifically, our experimental design extended the post-thawing incubation time at 38 °C up to 4 h, and two periods were split, the first from 0 to 2 h and the second from 2 to 4 h. Through this approach, the sequential effects of thawing on sperm function and DNA integrity could be evaluated in much detail, thus providing valuable information on the status of sperm cells when fertilization does not occur immediately after thawing. From the results obtained, one can conclude that the damage caused by ROS on sperm viability and motility occurs earlier than that observed on DNA, probably because ROS is not a determining factor leading to DNA fragmentation, but only induces DNA base modifications. Remarkably, the rate of increase for double-stranded breaks did not follow the same pattern than global DNA damage and had a marginal incidence, which differs from what reported in other species. These differences could be due to the fact that whereas all bulls included herein were fertile individuals, research conducted in humans gathers fertile and infertile men [36]. While, in this study, bovine sperm DNA appeared to be mainly affected by ROS, sperm from infertile men are known to present different types of DNA damage, which could not only arise from ROS but also from deficiencies in the activities of specific nucleases [37, 38]. We also assessed whether sperm DNA damage, either when considered at a specific time point (static) or when evaluated over the post-thawing period, was related to reproductive outcomes (NRR). Interestingly, the percentage of sperm with highly damaged DNA at 0 h was found to be negatively correlated with NRR.

### ROS is not responsible for the appearance of double-stranded breaks, but affects global DNA damage

Throughout the entire incubation period (0 to 4 h), the rate of increase for the percentage of sperm with total ROS correlated positively to that for the degree of global DNA damage assessed by the alkaline Comet. Based on this result, the more ROS generated, the higher the percentage of sperm with DNA damage. Contrarily, no correlation between ROS and the incidence of double-stranded breaks, as determined by the neutral Comet, either when considered at a specific time point (static) or when evaluated all over the incubation period, was observed. Because the alkaline Comet was performed after double helix denaturation, both single- and double-stranded DNA damage was measured [39]. It is worth noting that, as OTM results in Table 1 show, the incidence of double-stranded DNA breaks was much lower than that of global DNA damage (1.31 ± 0.2 vs. 15.57 ± 5.2, respectively, at 0 h). This suggests that, in cattle, most of the global sperm DNA damage detected by the alkaline Comet corresponds to single-stranded DNA breaks (SSB). While this observation is similar to that made in pigs [40], it differs from what reported in humans, where the proportion of double-stranded DNA breaks compared to that of single-stranded DNA breaks may be greater. In fact, increases of double-stranded DNA breaks in human sperm have previously been associated with men infertility and recurrent miscarriage [36]; these affectations, however, were not observed in the studied cattle cohort, as animals were of proven fertility.

Our results suggest that, although ROS are the main cause of SSB in bovine sperm, they may not lead to DSB. In comparison to other cell types, the metabolic rate of sperm at 37−38 °C is high, because their physiological processes require significant amounts of energy [41, 42]. This drives to a great generation of ROS, which are a byproduct that cause lipid peroxidation, protein alterations and DNA base modifications [43]. In the case of DNA, the most common base adduct is 8OHdG. Because sperm only have the first enzyme of the base excision repair mechanism, the 8-oxoguanosine DNA glycosylase (OGG1), there is no complete reparation and 8OHdG is removed leaving a single-stranded DNA break [23, 26].

Oxidative stress is a common detrimental feature of sperm from different species, and separate studies have shown that it leads to increased DNA damage [44–47]. Related to this, it is worth noting that the assessment of DNA fragmentation using acridine orange/sperm chromatin structure assay, *Terminal* deoxynucleotidyl transferase dUTP nick end labeling (TUNEL) assay or neutral Comet, which rely on different strategies to directly or indirectly infer the extent of DNA damage, may not always reflect the amount of single-stranded DNA breaks [48]. Furthermore, our results are agreement with a previous investigation that artificially induced oxidative DNA damage using hydrogen peroxide, evaluated that damage through both alkaline and neutral Comet assays and found that the outcomes provided by the alkaline Comet, but not those given by the neutral one [44], increased. In the light of our results, double-stranded breaks, despite being residual, would not mainly originate from ROS. Previous research has suggested that double-stranded breaks may be induced by an endogenous nuclease that cleaves sperm DNA in regions between toroids (TLR), which are linked to the nuclear matrix, are associated to histones rather than protamines, are less condensed and are thus more accessible to enzymes [49]. While double-stranded breaks could also be generated by other mechanisms, such as topoisomerases, which can induce permanent DSB if ATP is not hydrolyzed, whether this process also occurs when frozen-thawed sperm are subjected to post-thawing incubation is not clear [50–52]. Yet, any of these mechanisms would support the generation of a limited number of DSB and an increased incidence of DSB during the first period of our study, as nucleases would be activated upon thawing and their activity would be limited to toroid linker regions. Although DSB are present in low amounts in bovine sperm, their basal levels were found to be negatively correlated to sperm motility, suggesting that the greater the number of DSB, the higher the rate of motility reduction. Contrary to the absence of correlation between double-strand DNA damage and sperm motility in humans [2], our data point out to a possible relationship between DSB and sperm quality in cattle; this should be addressed in further studies.

### Sperm viability and sperm motility are the physiological parameters that are preferentially affected during the first period of incubation

**Fig. 3:**
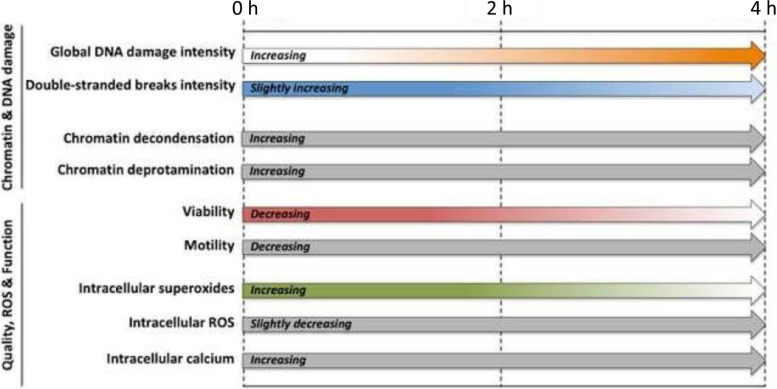
Schematic representation of the rates of increase/decrease for each parameter. Arrows in grey mean no variation in rates (increase or decrease), whereas colored arrows show how those rates were modified over post-thawing incubation time; the more intense the color, the higher the rate. Viability, double-stranded DNA breaks and intracellular superoxides exhibited a higher rate of decrease during the first period, whereas global DNA damage presented a higher rate of increase during the second period

Sperm viability was affected during the first post-thawing period, and sperm motility was constantly reduced throughout incubation. Interestingly, the variation rate of the percentage of motile sperm correlated positively to the rate of increase for global DNA damage, suggesting that the quicker the reduction in motility the less the rate of increase of DNA damage. In addition, total ROS levels, but not superoxide content, constantly decreased throughout incubation (Table 4). Although, at first glance, the combination of these two results could seem contradictory, they may indicate that, while all post-thaw injury effects (targeting viability, motility and DNA damage) are originated from the same oxidative stress mechanism, the detrimental impact on some physiological parameters, such as viability and motility, occurs earlier. Our data support that the ROS generated during freezing and thawing processes (before our 0 h time point) [53, 54] would oxidize lipids from mitochondrial and plasma membranes, leading to a rapid loss of mitochondrial membrane potential and plasma membrane integrity, which would ultimately abolish motility and induce cell death [10, 43]. Hence, the constant reduction of total ROS observed throughout post-thawing incubation (i.e., between 0 and 4 h) could be the consequence of a rapid reaction of these highly reactive species to cell components and the leakage of ROS from non-viable sperm cells. Moreover, our results support that function parameters such as viability and motility are altered faster than nuclear DNA breaks become apparent. In fact, as aforementioned, ROS may induce base modifications on sperm DNA which, via the activity of OGG1, lead to strand breaks [23, 26]. Because the high degree of sperm chromatin condensation in toroids could hinder OGG1 to access the site, it would be reasonable to assume that the DNA alterations caused by ROS could remain in the form of modified bases rather than DNA breaks, thus being undetectable by the methods evaluating DNA integrity. Our results support, therefore, that the first affectation of ROS after incubation of frozen-thawed bovine sperm targets viability and motility, the DNA damage only becoming apparent later (Fig. 3). Nonetheless, it remains unknown whether DNA base modifications are produced as plasma membrane and motility drop but remain silent, or if this is a three-stage process. Hence, further experiments assessing 8OHdG in comparison to the alkaline Comet assay in bovine sperm, or using a modified Comet assay including formamidopyrimidine DNA glycosylase (Fpg), which excises the modified nucleotides [55], would help refine this hypothesis.

Despite the major effect on motility and viability, other sperm function parameters, such as intracellular calcium, were also impaired during post-thawing incubation. In effect, a negative correlation between alkaline Comet DNA damage at 0 h and the increase rate of intracellular calcium was found, which would indicate that oxidative stress was responsible for both alterations. In fact, apart from causing DNA base oxidation, high ROS is known to promote sperm capacitation [56, 57]. In our case, a high degree of basal DNA breaks might suggest that many ROS would have already been used to produce DNA damage, so that less ROS would be available to cause an increase in intracellular calcium.

### Chromatin deprotamination, decondensation and DNA damage are related affectations

Proper chromatin condensation is crucial for sperm to protect their genetic material until they encounter the oocyte. At the same time, sperm chromatin must be able to decondense after fertilization, thus providing access to the male genome by the embryo machinery and allowing the formation of the paternal nucleus [58]. Recent investigations have shown that deprotamination in bovine sperm has a negative impact on their fertilizing capacity [59]. In the present work, we found that the rate of increase for double-stranded breaks during the first 2 h post-thawing was related to the extent of chromatin decondensation (Additional file 3: Table S2), and that the rise in global DNA damage was directly associated to chromatin deprotamination and decondensation during the second incubation period (Additional file 4: Table S3). Additionally, while the percentage of sperm with double-stranded breaks mostly increased during the first period, the amount of single-stranded breaks and the percentage of sperm with moderate or high damage occurred mostly during the second period (Table 1 and Fig. 1). In both periods, sperm decondensation and deprotamination were constant (Fig. 2). Previous research demonstrated that both removal of zinc through EDTA and oxidative stress lead to sperm deprotamination and decondensation [60, 61]. Based on our findings, one could posit that the second incubation period (2-4 h) allowed the access of OGG1 to the modified DNA bases, which resulted in the excision of nucleotides and generated single-stranded breaks. Under these post-thawing conditions, therefore, deprotamination and decondensation in specific chromatin regions caused by oxidative stress or other reducing agents could lead to the better access of OGG1. This would subsequently drive to the formation of single-stranded DNA breaks, and would be similar to the decondensation of sperm chromatin induced by DTT, which allows nuclease to degrade the DNA [37]. If this hypothesis was confirmed, chromatin deprotamination would represent an intermediate step between the impairment of sperm viability and motility and the generation of single-stranded DNA breaks (Fig. 3).

### Global DNA damage and not double-strand breaks negatively affect non-return rates

Improvement of breeding efficiency through the selection of the best bulls is a constant need for maximizing revenues in cattle industry. In this regard, and although selecting sires on the basis of their artificial insemination outcomes appears to be the best approach, it is obvious that it is not an efficient way of choosing animals, because: a) it is time consuming, and b) when reproductive data are available, the animal is older, and age is known to be detrimental on sperm quality [62]. In addition, it is well known that sperm functional parameters may change in relation to the season when semen is collected [63, 64], and this may generate a bias between samples used for sperm quality analysis and those used for fertilization. In our study, we could not obtain samples throughout the year, but we conducted analyses in pools of three ejaculates collected in 5-week periods, ensuring that each pool contained ejaculates obtained in at least two seasons. This made the pools more representative of sperm quality than a single ejaculate. On the basis of this design, we found that sperm motility was correlated to AI outcomes, which is in agreement with other studies in cattle that found an association between sperm motility, morphology and sire fertility [65, 66]. New approaches such as genetic characterization [67], functional analyses [67], or protein biomarkers [68] have been emerging in the last years as promising tools to predict sperm fertilizing capacity. Thus far, nevertheless, only a limited number of studies have focused on testing sperm chromatin as a biomarker for cattle fertility, some showing a relationship with DNA damage and fertility [28, 69, 70], and others reporting opposite results [71–73]. To the best of our knowledge, this is the first study assessing the relationship between the incidence of DSB/SSB in sperm and AI outcomes in cattle. Our results indicated that while DSB were not correlated to 90-d NRR, SSB – which were determined by the alkaline Comet – were negatively correlated to cattle fertility. The fact that only the percentage of sperm with high DNA damage, but not the one of sperm with moderate DNA damage, was negatively correlated to fertility could indicate that oocytes may have a limited capacity to repair paternal DNA. Thus, after fertilization, zygotes and embryos may be able to repair moderate, but not high, sperm DNA damage. In effect, sperm DNA damage has been suggested to impair in vitro embryo development in other species [2, 74–76], and embryo arrest in cattle may be an important cause of pregnancy loss, as only 50% of embryos are viable by 5-6 d post-insemination [77]. A recent study demonstrated that the most fertile cows have better follicle environment and oocyte quality [78], which supports that the oocyte quality may be related to the capacity of the embryo to repair the paternal DNA damage [79]. Additionally, we found that viable sperm with high ROS at 4 h post-thaw were correlated to NRR. As broadly discussed above, while oxidative stress is one of the major effectors leading to damage not only to paternal genetic content through base modifications, but also to different components of the sperm cell, such as plasma membrane, acrosome and proteins [43], ROS also drive sperm capacitation in small amounts [15, 16]. In cattle, previous studies suggested that highly imbalanced oxidative stress is directly related to fertility rates, both in vivo [80] and in vitro [81]. In our study, we found the ROS levels in sperm at 4 h post-thaw, which were present in low amounts, were positively correlated to bull fertility (Table 5). Because animals included in this study were of proven fertility, these data might suggest that low, rather than high, ROS amounts are needed for oocyte fertilization.

## Conclusions

In conclusion, the present study shows the sequence of post-thaw injury events occurring during incubation of frozen-thawed bovine sperm at 38 °C for 4 h. Interestingly, our data indicate that sperm viability and motility become affected earlier than DNA, and that the occurrence of single-stranded DNA breaks is concomitant to chromatin decondensation and deprotamination. Consequently, while sperm function parameters and single-stranded breaks share ROS as damaging agents, the occurrence of DNA breaks may need additional steps that are prevented by chromatin condensation – mainly the OGG1 activity –. Regarding the type of DNA breaks, the double-stranded ones are evidenced earlier than the single-stranded, possibly because of the activity of the different mechanisms involved (non-oxidative vs. oxidative, respectively). Finally, while no dynamic parameter was correlated to fertilization outcomes, the static analysis of global DNA damage through the alkaline Comet assay, sperm motility and intracellular ROS were found to be good predictors of bull fertility.

## Supplementary Information

**Additional file 1: Fig. S1.** Criteria used to distinguish (A) sperm with normal decondensation, and (B) sperm with high chromatin decondensation (Bar = 10 μm).

**Additional file 2: Table S1.** Correlations between sperm DNA fragmentation, chromatin decondensation and sperm quality and functionality parameters throughout 4 h of incubation.

**Additional file 3: Table S2.** Correlations between sperm DNA fragmentation, chromatin decondensation and sperm quality and functionality parameters for the first period of incubation.

**Additional file 4: Table S3.** Correlations between sperm DNA fragmentation, chromatin decondensation and sperm quality and functionality parameters for the second period of incubation.

## Data Availability

The datasets used and/or analyzed during the current study are available from the corresponding author on reasonable request.

## Abbreviations

8OHdG: 8′-hydroxy-2'deoxyguanosine
AI: Artificial insemination
ALH: Mean amplitude of lateral head displacement
AU: Arbitrary units
BCF: Frequency of head displacement
CMA3: Chromomycin
DCF: Dichlorofluorescein
DSB: Double-stranded DNA breaks
DTT: Dithiothreitol
E: Ethidium
FITC: Fluorescein isothiocyanate
HE: Hydroethidine
LIN: Linearity coefficient
NRR: 90-d non-return rates to estrus
OGG1: 8-oxoguanosine DNA glycosylase
OS: Oxidative stress
OTM: Olive tail moment
PCA: Principal component analysis
PI: Propidium Iodide
ROS: Reactive oxygen species
SSB: Single-stranded DNA breaks
STR: Straightness coefficient
TUNEL: Terminal deoxynucleotidyl transferase dUTP nick end labeling
VAP: Average path velocity
VCL: Curvilinear velocity
VSL: Straight-line velocity
WOB: Wobble coefficient
