# Supplementary figures and images for: Determination of double- and single-stranded DNA breaks in bovine sperm is predictive of their fertilizing capacity
Source: J Anim Sci Biotechnol. 2022 Sep 17;13:105. doi: 10.1186/s40104-022-00754-8 (PMC9482281; doi:10.1186/s40104-022-00754-8)

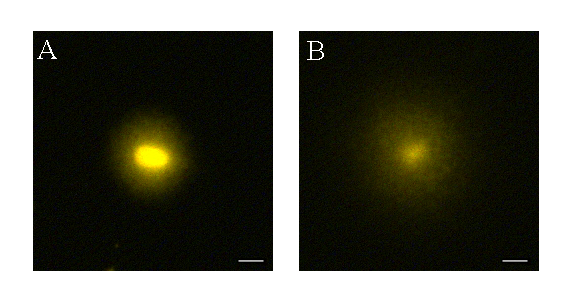

Supplement: Supplementary file 1 — Additional file 1: Fig. S1. Criteria used to distinguish (A) sperm with normal decondensation, and (B) sperm with high chromatin decondensation (Bar = 10 μm). [file 40104_2022_754_MOESM1_ESM.tif]
