# Supplementary material for: Determination of double- and single-stranded DNA breaks in bovine sperm is predictive of their fertilizing capacity
Source: J Anim Sci Biotechnol. 2022 Sep 17;13:105. doi: 10.1186/s40104-022-00754-8 (PMC9482281; doi:10.1186/s40104-022-00754-8)
Supplement: Supplementary file 2 — Additional file 2: Table S1. Correlations between sperm DNA fragmentation, chromatin decondensation and sperm quality and functionality parameters throughout 4 h of incubation. [file 40104_2022_754_MOESM2_ESM.docx]

| **Additional File 2: Table S1**. Correlations between sperm DNA fragmentation, chromatin decondensation and sperm quality and functionality parameters throughout 4 hours of post-thawing incubation.  OTM: Olive tail moment, indicating DNA breaks intensity; %SDF: Percentage of sperm DNA fragmentation, indicating the percentage of fragmented cells | | | | | | | | | | | | |
| --- | --- | --- | --- | --- | --- | --- | --- | --- | --- | --- | --- | --- |
|  |  | Viability, ΔT4-T0 | Progressive motility, ΔT4-T0 | Total motility, ΔT4-T0 | %Fast sperm, ΔT4-T0 | %DNA decon-densation, ΔT4-T0 | Poor protami-nation intensity, ΔT4-T0 | Poor protami-nation, %, ΔT4-T0 | Intracellular ROS, DCF^+^,  ΔT4-T0 | Intracellular superoxides, E^+^,  ΔT4-T0 | Intracellular calcium, F3^+^,  ΔT4-T0 |  |
| Alkaline Comet OTM, T0 | *Rs* | 0.200 | -0.196 | -0.116 | -0.168 | -0.179 | -0.035 | -0.097 | -0.107 | -0.054 | **-0.460^*^** |  |
|  | *P*-value | 0.339 | 0.347 | 0.580 | 0.422 | 0.391 | 0.867 | 0.643 | 0.646 | 0.797 | 0.036 |  |
| Alkaline Comet OTM, ΔT4-T0 | *Rs* | 0.032 | 0.010 | -0.124 | 0.035 | **0.430^*^** | 0.125 | 0.061 | 0.305 | -0.239 | 0.030 |  |
|  | *P*-value | 0.878 | 0.962 | 0.555 | 0.867 | 0.032 | 0.550 | 0.773 | 0.179 | 0.250 | 0.898 |  |
| Alkaline Comet %SDF Moderate + High, T0 | *Rs* | 0.192 | -0.251 | -0.144 | -0.217 | -0.202 | -0.003 | -0.149 | -0.185 | -0.050 | **-0.512^*^** |  |
|  | *P*-value | 0.359 | 0.226 | 0.493 | 0.298 | 0.334 | 0.987 | 0.479 | 0.422 | 0.811 | 0.018 |  |
| Alkaline Comet %SDF Moderate + High, ΔT4-T0 | *Rs* | 0.092 | -0.020 | -0.053 | 0.023 | 0.286 | -0.134 | -0.061 | **0.604^*^** | -0.325 | -0.166 |  |
|  | *P*-value | 0.661 | 0.924 | 0.801 | 0.913 | 0.166 | 0.524 | 0.773 | 0.004 | 0.113 | 0.471 |  |
| Neutral Comet OTM, T0 | *Rs* | -0.089 | -0.329 | -0.227 | -0.333 | -0.315 | -0.143 | -0.238 | 0.081 | 0.190 | -0.015 |  |
|  | *P*-value | 0.672 | 0.109 | 0.276 | 0.104 | 0.125 | 0.496 | 0.253 | 0.728 | 0.363 | 0.949 |  |
| Neutral Comet OTM, ΔT4-T0 | *Rs* | 0.073 | 0.029 | -0.065 | 0.188 | 0.088 | -0.332 | -0.210 | 0.029 | 0.219 | 0.104 |  |
|  | *P*-value | 0.727 | 0.891 | 0.756 | 0.369 | 0.677 | 0.105 | 0.313 | 0.902 | 0.293 | 0.654 |  |
| Neutral Comet %SDF, T0 | *Rs* | -0.311 | 0.057 | 0.170 | 0.000 | 0.340 | 0.000 | 0.142 | 0.000 | 0.241 | 0.000 |  |
|  | *P*-value | 0.130 | 0.788 | 0.417 | 1.000 | 0.097 | 1.000 | 0.500 | 1.000 | 0.247 | 1.000 |  |
| Neutral Comet %SDF, ΔT4-T0 | *Rs* | 0.186 | -0.312 | -0.392 | -0.183 | -0.334 | -0.272 | -0.337 | -0.146 | 0.226 | 0.156 |  |
|  | *P*-value | 0.375 | 0.128 | 0.053 | 0.382 | 0.103 | 0.188 | 0.100 | 0.529 | 0.277 | 0.501 |  |
| ^*^Indicate statistically significant correlations |  |  |  |  |  |  |  |  |  |  |  |  |
