# Supplementary material for: Determination of double- and single-stranded DNA breaks in bovine sperm is predictive of their fertilizing capacity
Source: J Anim Sci Biotechnol. 2022 Sep 17;13:105. doi: 10.1186/s40104-022-00754-8 (PMC9482281; doi:10.1186/s40104-022-00754-8)
Supplement: Supplementary file 3 — Additional file 3: Table S2. Correlations between sperm DNA fragmentation, chromatin decondensation and sperm quality and functionality parameters for the first period of incubation. [file 40104_2022_754_MOESM3_ESM.docx]

| **Additional File 3: Table S2**. Correlations between sperm DNA fragmentation, chromatin decondensation and sperm quality and functionality parameters for the first period of incubation.  OTM: Olive tail moment, indicating OTM; %SDF: Percentage of sperm DNA fragmentation, indicating the percentage of fragmented cells | | | | | | | | | | | | |
| --- | --- | --- | --- | --- | --- | --- | --- | --- | --- | --- | --- | --- |
|  |  | Viability, ΔT2-T0 | Progressive motility, ΔT2-T0 | Total motility, ΔT2-T0 | %Fast sperm, ΔT2-T0 | %DNA decon-densation, ΔT2-T0 | Poor protami-nation intensity, ΔT2-T0 | Poor protami-nation, %, ΔT2-T0 | Intracellular ROS, DCF^+^,  ΔT2-T0 | Intracellular superoxides, E^+^,  ΔT2-T0 | Intracellular calcium, F3^+^,  ΔT2-T0 |  |
| Alkaline Comet OTM, T0 | *Rs* | -0.296 | -0.046 | 0.079 | -0.060 | -0.226 | -0.150 | -0.207 | -0.069 | 0.037 | -0.339 |  |
|  | *P*-value | 0.151 | 0.853 | 0.748 | 0.808 | 0.277 | 0.474 | 0.322 | 0.767 | 0.861 | 0.133 |  |
| Alkaline Comet OTM, ΔT2-T0 | *Rs* | 0.165 | -0.133 | -0.284 | -0.096 | 0.350 | 0.228 | 0.116 | -0.073 | 0.248 | 0.321 |  |
|  | *P*-value | 0.430 | 0.586 | 0.238 | 0.694 | 0.086 | 0.274 | 0.580 | 0.754 | 0.231 | 0.156 |  |
| Alkaline Comet %SDF Moderate + High, T0 | *Rs* | -0.184 | -0.109 | 0.057 | -0.096 | -0.262 | -0.152 | -0.266 | -0.012 | 0.039 | -0.348 |  |
|  | *P*-value | 0.380 | 0.657 | 0.816 | 0.697 | 0.205 | 0.469 | 0.198 | 0.958 | 0.854 | 0.123 |  |
| Alkaline Comet %SDF Moderate + High, ΔT2-T0 | *Rs* | 0.024 | **0.484^*^** | **0.475^*^** | 0.361 | 0.033 | 0.162 | 0.153 | -0.422 | -0.193 | -0.224 |  |
|  | *P*-value | 0.910 | 0.036 | 0.040 | 0.128 | 0.877 | 0.439 | 0.464 | 0.057 | 0.356 | 0.329 |  |
| Neutral Comet OTM, T0 | *Rs* | 0.131 | **-0.473^*^** | -0.257 | -0.396 | -0.283 | -0.282 | -0.266 | 0.193 | 0.015 | 0.053 |  |
|  | *P*-value | 0.531 | 0.041 | 0.287 | 0.093 | 0.171 | 0.173 | 0.199 | 0.403 | 0.945 | 0.821 |  |
| Neutral Comet OTM, ΔT2-T0 | *Rs* | 0.022 | 0.021 | -0.118 | 0.060 | **0.477^*^** | 0.179 | 0.260 | 0.090 | 0.104 | 0.322 |  |
|  | *P*-value | 0.919 | 0.932 | 0.632 | 0.808 | 0.016 | 0.393 | 0.209 | 0.699 | 0.621 | 0.154 |  |
| Neutral Comet %SDF, T0 | *Rs* | -0.311 | -0.215 | 0.000 | -0.301 | -0.085 | -0.085 | 0.340 | 0.000 | 0.113 | 0.000 |  |
|  | *P*-value | 0.130 | 0.376 | 1.000 | 0.210 | 0.687 | 0.687 | 0.097 | 1.000 | 0.590 | 1.000 |  |
| Neutral Comet %SDF, ΔT2-T0 | *Rs* | -0.089 | 0.056 | -0.330 | 0.070 | 0.032 | 0.037 | -0.129 | -0.003 | 0.087 | 0.117 |  |
|  | *P*-value | 0.671 | 0.819 | 0.167 | 0.775 | 0.881 | 0.859 | 0.540 | 0.989 | 0.679 | 0.614 |  |

^*^indicate statistically significant correlations
