# Supplementary material for: Determination of double- and single-stranded DNA breaks in bovine sperm is predictive of their fertilizing capacity
Source: J Anim Sci Biotechnol. 2022 Sep 17;13:105. doi: 10.1186/s40104-022-00754-8 (PMC9482281; doi:10.1186/s40104-022-00754-8)
Supplement: Supplementary file 4 — Additional file 4: Table S3. Correlations between sperm DNA fragmentation, chromatin decondensation and sperm quality and functionality parameters for the second period of incubation. [file 40104_2022_754_MOESM4_ESM.docx]

| **Additional File 4: Table S3**. Correlations between sperm DNA fragmentation, chromatin decondensation and sperm quality and functionality parameters for the second period of incubation.  OTM: Olive tail moment, indicating DNA breaks intensity; %SDF: Percentage of sperm DNA fragmentation, indicating the percentage of fragmented cells | | | | | | | | | | | | |
| --- | --- | --- | --- | --- | --- | --- | --- | --- | --- | --- | --- | --- |
|  |  | Viability, ΔT4-T2 | Progressive motility, ΔT4-T2 | Total motility, ΔT4-T2 | %Fast sperm, ΔT4-T2 | %DNA decon-densation, ΔT4-T2 | Poor protami-nation intensity, ΔT4-T2 | Poor protami-nation, %, ΔT4-T2 | Intracellular ROS, DCF^+^,  ΔT4-T2 | Intracellular superoxides, E^+^,  ΔT4-T2 | Intracellular calcium, F3^+^,  ΔT4-T2 |  |
| Alkaline Comet OTM, T0 | *Rs* | 0.290 | -0.158 | 0.026 | 0.011 | 0.000 | 0.120 | 0.096 | 0.207 | -0.131 | -0.126 |  |
|  | *P*-value | 0.159 | 0.519 | 0.915 | 0.965 | 1.000 | 0.566 | 0.649 | 0.321 | 0.533 | 0.548 |  |
| Alkaline Comet OTM, ΔT4-T2 | *Rs* | 0.285 | -0.046 | 0.027 | -0.077 | 0.335 | **0.607^*^** | 0.312 | 0.039 | **-0.415^*^** | 0.078 |  |
|  | *P*-value | 0.167 | 0.853 | 0.910 | 0.748 | 0.101 | 0.001 | 0.130 | 0.852 | 0.039 | 0.709 |  |
| Alkaline Comet %SDF Moderate + High, T0 | *Rs* | 0.219 | -0.230 | 0.023 | -0.004 | 0.031 | 0.150 | 0.141 | 0.137 | -0.155 | -0.193 |  |
|  | *P*-value | 0.294 | 0.344 | 0.922 | 0.987 | 0.882 | 0.473 | 0.501 | 0.514 | 0.458 | 0.356 |  |
| Alkaline Comet %SDF Moderate + High, ΔT4-T2 | *Rs* | 0.332 | -0.074 | -0.211 | -0.268 | **0.468^*^** | **0.425^*^** | 0.181 | 0.304 | -0.313 | -0.187 |  |
|  | *P*-value | 0.105 | 0.764 | 0.373 | 0.254 | 0.018 | 0.034 | 0.387 | 0.140 | 0.128 | 0.371 |  |
| Neutral Comet OTM,T0 | *Rs* | -0.202 | -0.272 | -0.126 | -0.109 | -0.036 | 0.055 | 0.133 | -0.125 | 0.154 | 0.071 |  |
|  | *P*-value | 0.333 | 0.259 | 0.597 | 0.647 | 0.864 | 0.795 | 0.525 | 0.552 | 0.462 | 0.735 |  |
| Neutral Comet OTM, ΔT4-T2 | *Rs* | 0.162 | -0.338 | -0.391 | -0.308 | 0.096 | 0.187 | 0.122 | 0.163 | -0.112 | -0.304 |  |
|  | *P*-value | 0.438 | 0.157 | 0.089 | 0.187 | 0.647 | 0.372 | 0.560 | 0.435 | 0.594 | 0.139 |  |
| Neutral Comet %SDF, T0 | *Rs* | -0.113 | 0.172 | 0.219 | 0.099 | 0.340 | 0.028 | -0.340 | -0.297 | 0.255 | 0.085 |  |
|  | *P*-value | 0.590 | 0.481 | 0.354 | 0.677 | 0.097 | 0.893 | 0.097 | 0.149 | 0.219 | 0.687 |  |
| Neutral Comet %SDF, ΔT4-T2 | *Rs* | 0.074 | 0.137 | -0.030 | 0.021 | 0.193 | -0.012 | -0.015 | 0.162 | 0.203 | -0.265 |  |
|  | *P*-value | 0.725 | 0.577 | 0.899 | 0.930 | 0.355 | 0.953 | 0.943 | 0.440 | 0.330 | 0.201 |  |

*Indicate statistically significant correlation
